# Cephalometric and Photographic Evaluation of the Nasolabial Angle in Orthodontically Treated Patients: An Observational Cohort Study

**DOI:** 10.3390/diagnostics15020132

**Published:** 2025-01-08

**Authors:** Silvia Izabella Pop, Eugen Bud, Krisztina Mártha, Izabella Éva Mureșan, Kinga Mária Jánosi, Boglárka Dósa, Bernadette Kerekes-Máthé

**Affiliations:** Faculty of Dental Medicine, George Emil Palade University of Medicine, Pharmacy, Science, and Technology of Targu Mures, 38 Gh. Marinescu Str., 540139 Targu Mures, Romania; silvia.pop@umfst.ro (S.I.P.); krisztina.martha@umfst.ro (K.M.); izabella-eva.muresan@umfst.ro (I.É.M.); kinga.janosi@umfst.ro (K.M.J.); dosabeabglarka@gmail.com (B.D.); bernadette.kerekes-mathe@umfst.ro (B.K.-M.)

**Keywords:** nasolabial angle, orthodontics, soft tissue, upper lip thickness

## Abstract

**Background:** The nasolabial angle (NLA) is one of the most critical parameters of the soft tissue profile when orthodontic treatment is required. The primary aim of this prospective cohort study was to compare the differences in the evaluation made on lateral photographs and cephalograms. The secondary aim was to evaluate the modifications of the NLA (nasal and labial components) after orthodontic treatment, including upper first premolar extraction. **Methods:** The pre- and post-treatment lateral radiographs and profile photographs of 60 subjects (18 male and 42 female) treated with upper premolar extraction and fixed orthodontic appliances were evaluated. The nasolabial angle was measured in both investigations, while cephalometric parameters (skeletal, dental, and soft tissue parameters) were evaluated using lateral radiographs. **Results:** No statistically significant difference was observed between the results of the two types of measurements on the photographs and radiographs. The soft tissue parameters (ULT and UL-e) showed significant changes after orthodontic treatment, with ULT changing from 21.4 mm ± 4.07 mm to 22.9 mm ± 4.06 mm (*p* = 0.03) and UL-e changing from 8.42 mm ± 4.84 mm to 10.35 mm ± 4.23 mm (*p* < 0.001). In patients with thinner lips, the upper lip repositioning was more significant (*p* = 0.001). No statistically significant difference (*p* = 0.67) was found between the two evaluation methods. **Conclusions:** Both cephalometric and photographic evaluations of the nasolabial angle were similar. The soft tissue parameters (ULT and UL-e) showed significant changes after the orthodontic treatment. The repositioning of the upper lip was more acute when the lip thickness decreased.

## 1. Introduction

One of the main objectives of orthodontic treatment is achieving an aesthetic profile and balanced soft tissue contours. The nasolabial angle (NLA) is one of the most critical parameters of the soft tissue profile. Fradeani [1] defines the nasolabial angle as follows: an angle created by the intersection of two straight lines at the level of the subnasale point (Sn). One line is the tangent to the base of the nose (Columella nasi—Cm), and the other is the tangent to the outer edge of the upper lip (Ls) [1].

Regarding the normal value of the nasolabial angle, in the scientific literature, there is a significant difference between the sexes [2]. Fradeani et al. described 100–105° to be ideal for women and 90–95° to be ideal for men [1]. Bergman et al. determined an average value of 102° ± 8° [2]. Fernandez-Riveiro et al. determined a normal mean value of 105° ± 13° for women and 107.6° ± 8.5° for men [3]. This also suggests that the ethnicity of the subjects participating in the research may influence the results [4]. Another factor that may affect the measurements is the observed differences in the method of determining the subnasale point [5].

The nasolabial angle plays a vital role in the aesthetics of the facial profile, and its value may limit the treatment options, especially when a tooth extraction decision is required [6]. Tooth extraction for orthodontic purposes is a controversial topic in orthodontics, and according to the literature, it significantly impacts soft tissue aesthetics [6,7]. In general, the value of the nasolabial angle increases due to the retruded position of the lips [7,8]. In a survey by Talass et al., in subjects with class II malocclusion who had premolars removed as part of the treatment, the upper incisors retruded by an average of 6.7 mm, and the nasolabial angle increased by 10.5° (1.6 degrees per millimeter) [9].

Rathod et al. investigated the long-term soft tissue profile changes in an extraction sample compared with an untreated sample [10]. They concluded that the soft tissue profiles of the extraction sample had measurable changes in the lips and the chin [10].

The inclination of the upper incisors and the upper lip’s thickness indirectly affect the nasolabial angle’s labial component [11,12,13,14]. The incisors’ buccal-lingual movement directly affects the support of the lips [12]. The literature points to a general agreement that the inclination of the upper incisors is related to changes in the soft tissue, thus affecting the aesthetics of the face [13]. However, the upper lip’s modifications after incisor retraction are related to the upper lip’s initial thickness [14].

The profile analysis can be performed on a lateral photograph of the patient or on a lateral cephalogram. Each method has several advantages and drawbacks, mainly related to the head position and the reference lines used [15,16,17,18]. Therefore, we consider useful, from a clinical standpoint, a study comparing the cephalometric and photographic evaluation of the nasolabial angle. Both methods are used by orthodontists during case study preparations, and comparing the two investigations might add valuable information to the diagnostic.

The primary aim of this retrospective study is to compare the differences in the evaluation made on lateral photographs and cephalogram. The secondary aim is to evaluate the modifications of the nasolabial angle (both nasal and labial components) after orthodontic treatment, including upper first premolar extractions.

## 2. Materials and Methods

This present study was performed in accordance with the Declaration of Helsinki. The Scientific Research Ethics Committee of the George Emil Palade University of Medicine, Pharmacy, Science, and Technology in Târgu Mures approved the design and the consent forms of the present study (approval no. 3264/25.06.2024). All patients signed a written informed consent for participation in this study. Lateral radiographs and profile photographs of patients treated at the Orthodontic Department of the Dental Medicine Faculty of “George Emil Palade” University of Medicine, Pharmacy, Science, and Technology of Târgu Mureș and the Natural Smile Dental Clinic were examined.

### Study Design, Patient Selection, and Measurements

The sample size was determined using G*Power version 3.1.9.7 software (Franz Faul, Universität Kiel, Kiel, Germany). The calculations indicated that a minimum of 46 samples would be necessary; this size would provide a power greater than 95% that would detect significant differences, with an effect size of 0.8 at a significance level of α = 0.05.

The sample consisted of 60 subjects—18 male and 42 female patients—with a mean age of 19.6 (minimum age 16.2 and maximum age 26.9). The inclusion criteria for this study were as follows:

- White female and male healthy patients;
- No history of orthodontic treatment or facial surgery;
- Upper premolar extraction;
- Radiographs showing good hard and soft tissue resolution;
- Good quality and contrast imaging.

The patients were treated with fixed orthodontic appliances after with upper first or second premolar extraction. Roth bracket prescriptions were used (American Orthodontics, Sheboygan, WI, USA).

The exclusion criteria were as follows:

- Previous orthodontic treatment:
  - History of trauma;
  - Surgical interventions at the level of head or neck;
- The presence of congenital facial deformities;
- Low X-ray image quality;
- Movement or metal artifacts on the lateral radiographs.

In each of the selected cases, based on the diagnostic process, the upper premolars were removed as an elective space-creating method for the treatment. The sample included a wide spectrum of profile types, including protruded or retruded upper lip and nose morphology in patients with angle class I, II, and III dental and skeletal anomalies.

Lateral radiographs were captured using Pax Flex 3D+, a Vatech X-ray machine with an exposure time of 12.9 s, 80 kVp, and 9.0 mA. Measurements on the pre-treatment and post-treatment lateral radiographs and profile photographs were made digitally using orthodontic diagnostic software (Romexis 6.0.1, Planmeca OY, Helsinki, Finland). Profile pictures of the patients were captured using a Nikon D7500 camera ( Nikon Corporation, Tokyo, Japan) and 100 macro lenses, illuminated by a ring flash.

**Figure 1:**
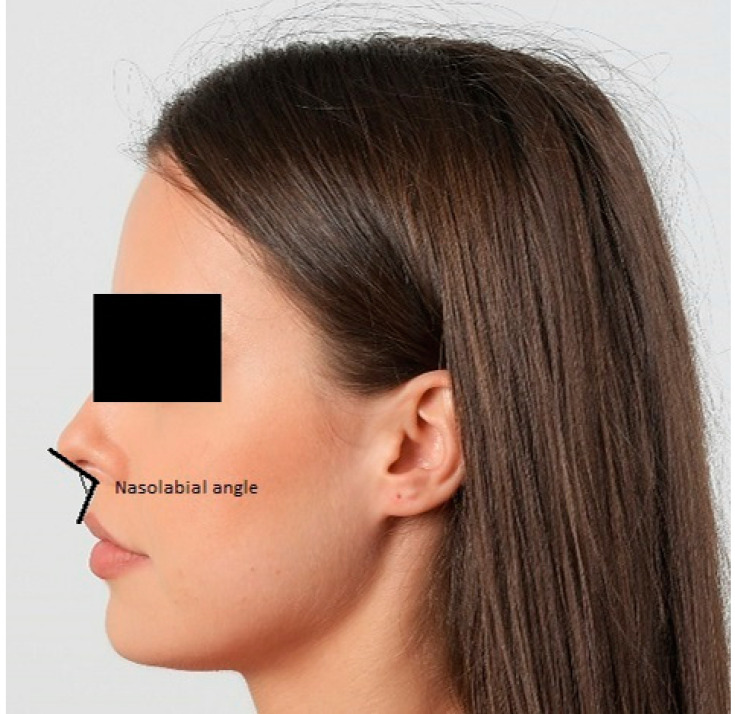
Nasolabial angle measurements on the profile photograph: tangent drawn from the subnasale (Sn) point to the philtrum of the upper lip through the labral superior (Ls) point and to the nose through the columella nasi (Cm) point.

From the methodological point of view, the two components of the nasolabial angle (NLA) (tangent drawn from the subnasale (Sn) point to the philtrum of the upper lip through the labral superior (Ls) point and to the nose through the columella nasi (Cm) point) were measured. For an initial assessment, the first measurement of the nasolabial angle was performed on the photographs using the software’s soft tissue analysis program (Romexis 6.0.1, Planmeca OY, Helsinki, Finland). In the photographs, the columella nasi (Cm), subnasale (Sn), and labral superior (Ls) points were marked manually. Based on the marked points, the software automatically measured the angle values. This measurement was named NLA_1 (Figure 1).

The second measurement of the nasolabial angle was performed on the lateral X-ray image, which was made in natural head position. For this purpose, a personalized cephalometric analysis was made. The recordings were calibrated using a millimeter scale, and the Frankfurt horizontal plane was adjusted to the horizontal plane. After calibration, the cephalometric points were marked on the radiographs (Table 1). The landmarks and their definitions were used according to Bergmans et al.’s recommendations [4].

**Figure 2:**
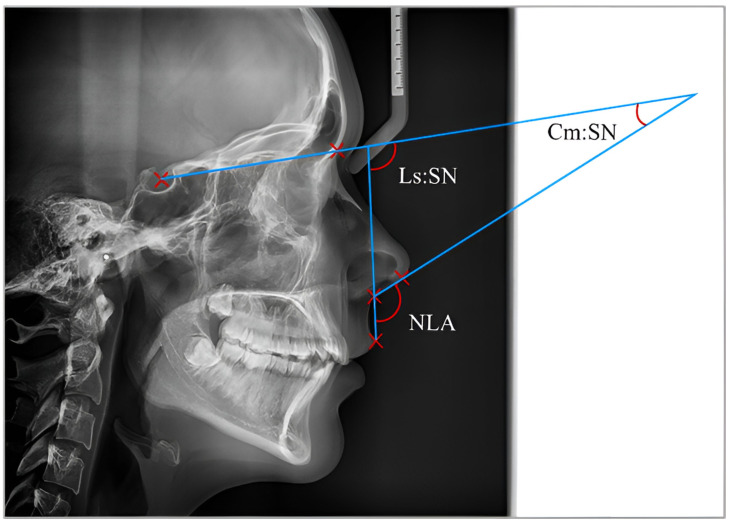
Nasolabial angle measurements on the lateral radiograph: the tangent of the base of the nose to the SN plane and the tangent of the upper lip to the SN formed two angles, Cm:SN (columella:sella–nasion angle) and Ls:SN (labrale superior:sella–nasion).

The following reference lines were used to draw the nasolabial angle (NLA_2) on the radiographs: the tangent of the base of the nose to the SN plane and the tangent of the upper lip to the same plane (SN). These lines formed two angles named Cm:SN (columella:sella–nasion angle), and Ls:SN (labrale superior:sella–nasion angle) (Figure 2).

The nasolabial angle’s upper lip and nose pad components were evaluated separately and independently.

Dividing the angle into two components is therefore important, as the values obtained in this way provide information on where the deviation can be derived from, i.e., to what extent the change is due to the position of the nasal bridge and to what extent can it be attributed to the position of the upper lip in each case. Since only the upper lip component can be affected by orthodontic intervention, parameters that are related to the upper lip and the lower face were selected:

Skeletal parameters:

- SNA angle—indicates the position of the maxilla in the sagittal plane. We considered it essential to examine because the maxilla provides bony support for the upper lips;
- SN-P angle—shows the position between the skull base and the bispinal line (palatal plane—PP), i.e., the vertical position of the maxilla;
- ANS-Gn—shows the distance between the spina nasalis anterior and the bony gnathion, i.e., the skeletal height of the lower third of the face;
- GoGn-SN—provides information about the position of the base of the mandible [5].

Dental parameters:

- Is-SN—the angle formed by the axis of the upper incisors and the base of the skull shows the sagittal position of the incisors in relation to the base of the skull and independently of the maxilla;
- Is-PP—the angle between the axis of the upper incisors and the bispinal line (palatal plane—PP), showing the position of the incisors in relation to the vertical position of the maxilla;
- Is-NA—the angle enclosed by the line between the axis of the upper incisors and the nasion, A-point, showing the position of the incisors in relation to the sagittal position of the maxilla;
- Is-NA—the distance between the most incisally located point of the upper incisors and the NA-line, expressed in millimeters [5].

Soft tissue parameters:

Since the nasolabial angle is the angle between soft tissue points, we considered it relevant to select soft tissue parameters related to the underlying skeletal and dental factors. These are

- ULT (upper lip thickness)—upper lip thickness, which is the distance between the outer and inner points of the upper lip (Ls-Ls_int);
- UL-E—the distance of the upper lip from the E-line (Rickkets’ aesthetic line drawn between the pronasale and soft tissue pogonion points).

The results were subjected to statistical analysis. No outliers were found after using the Grubbs test to identify them. Given the normal distribution of the data, as confirmed using the Shapiro–Wilk test, a paired *t*-test was chosen to assess the difference between the values obtained before and after treatment, and the Pearson test was chosen to identify the correlation between the study parameters. The mean (M), standard deviation (SD), and Pearson’s correlation coefficient (r) were calculated. The statistical significance was set at *p* < 0.05.

## 3. Results

No statistically significant difference was observed between the results of the two types of measurements on the photographs (NLA_1) and radiographs (NLA_2) (*p* = 0.67) (Table 2). However, the nasolabial angle showed an increasing trend in both measurements after the treatment. No statistically significant difference was seen between the male and female patients.

**Figure 3:**
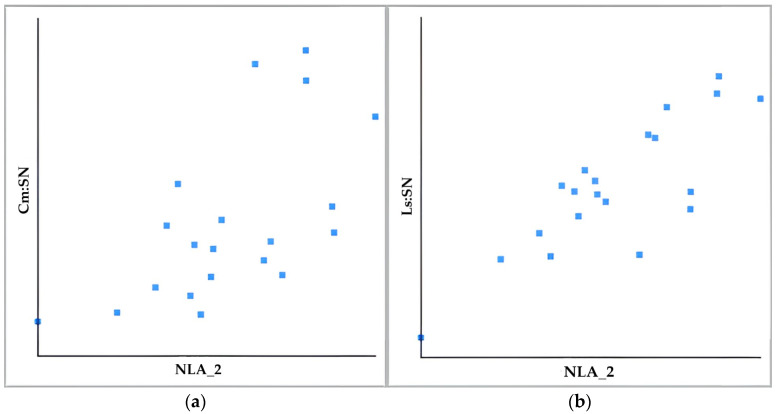
Diagrams showing the correlation (**a**) between Cm:SN (Y) and NLA_2 (X) (r = 0.61) and (**b**) between Ls:SN (Y) and NLA_2 (X) (r = 0.82).

The Pearson’s correlation coefficient results showed that changes in the upper lip component are more closely related to changes in the nasolabial angle than changes in the position of the nasal tip (r greater than 0.5 but less than 0.7) (Figure 3).

The skeletal, dental, and soft tissue parameter changes are shown in Table 3.

One of the soft tissue parameters, UL-E, showed the most significant change: a difference of 22.92%. This was followed by Is-NA, with a 17.98% change, and then ULT, which deviated from its original position by 7%. The GoGn-N angle changed the least, by 0.63%. The variation in the other parameters is between 0.95% and 3.47%.

**Figure 4:**
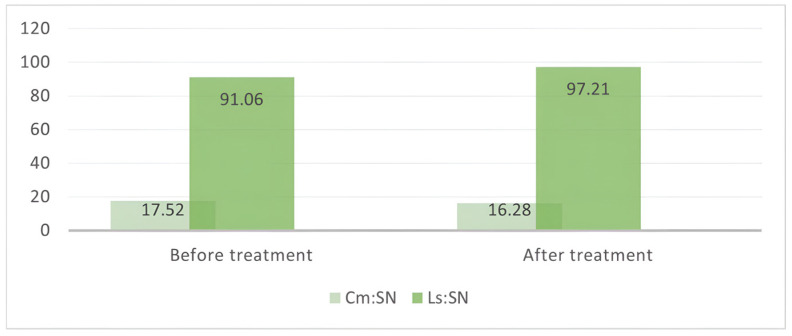
The modifications of Cm:SN and Ls:SN before and after the orthodontic treatment.

As shown in Figure 4, the position of the upper lip showed a significant increase (*p* < 0.05).

We subjected the soft tissue parameters to further observations since they showed significant differences. The research samples were divided into two groups according to upper lip thickness: the first group consisted of values below the average value (21.4 mm), and the second group consisted of values above the average. Additional paired *t*-tests were performed. The individuals with thinner lips showed a significant difference after the treatment (*p* = 0.001), while the changes in the second group were insignificant (*p* = 0.71).

## 4. Discussion

The nasolabial angle is a strategic point in profile aesthetics when planning orthodontic treatment. In recent years, several studies [3,4,5,6,7,8,9,10,11,19,20,21,22,23,24,25,26,27,28,29,30] have examined the influence of the nasolabial angle on profile aesthetics. The data in the literature are controversial regarding the mean ideal value of the nasolabial angle. Fitzgerald et al. [5] determined a mean value of 114.08° ± 9.58°, and Gołębiowski et al. [8] determined a mean value of 114.07° ± 10.81°. The variation in the nasolabial angle also depends on gender and initial malocclusion. Perović et al. [18] concluded, in their study, that the difference between angle classes was not significant. Ballin et al. [19] determined the average values of NLA according to gender: 107.75° ± 9.82°for men and 104.03° ± 10.65° for women. In our study, no significant changes were obtained between the male and female patients.

Nasolabial angle also varies according to the racial characteristics of the evaluated individuals. Regarding the ethnic grouping, for example, Uysai et al. [20] measured the nasolabial angle mean values in a group of North American subjects and compared them to a group of Turkish subjects. Our results are like those of the abovementioned study. The mean value of the angle was 111.61° ± 11.97°. When comparing the values of the nasolabial angle before and after the orthodontic treatment, our study showed no significant difference. However, there is a tendency for the nasolabial angle to decrease after orthodontic treatment. Our findings are in accordance with the outcome of other similar studies in the literature [22,30].

In their study, Bravo et al. [30] worked with 31 subjects with angle class II malocclusion, of which 15 received non-extraction and 16 received extraction orthodontic treatment. They concluded that the significant soft tissue differences between the groups at the end of treatment were a more retruded lower lip and a more pronounced lower labial sulcus. In another study, Kocadereli [22] studied 40 extraction treatments with angle class I anomalies, with four-premolar extraction. Their results showed an increased nasolabial angle and more retruded upper and lower lips in the extraction patients. In a similar survey conducted by Ismail et al. [31], the subjects in the extraction group exhibited an increase in the concavity of the labiomental fold. In contrast, the non-extraction group showed no changes in that area. Their assessment was performed three-dimensionally, while a two-dimensional cephalometric analysis was performed in our study.

The research conducted by Stephens et al. [32] was similar, from a methodological point of view, to our study. They evaluated several soft tissue cephalometric parameters in non-extraction and extraction groups of patients. Holdaway’s H-line was used as a reference. Their results showed that, in the long term, the treatment modality does not affect long-term soft tissue profile changes. In our study, both hard and soft tissue changes were evaluated. For the skeletal parameters, the SNA angle was used to assess the position of the maxilla, while for the dental parameters, the position of the upper incisors was used (Is:SN, Is:PP, Is:NA, and Is-NA). We did not find any significant changes in the dental parameters between the pre-treatment and post-treatment states.

The parameters of the upper lip showed significant changes. Both the thickness of the upper lip and its position relative to the E-line were changed. The increase in the upper lip thickness can be explained in terms of the lip strain. When the upper incisors are protruded, a significant muscular strain may be present in the upper lip [21,33]. This strain might give the impression of a thinner upper lip. Modifying the upper incisor proclination reduces the muscular tension, and the lip appears to be thicker. Another explanation for the modified upper lip parameters is related to the initial lip thickness. Holdaway stated in his paper [34] that the thicker the upper lip is, the less repositioning will occur when the upper incisors are retruded. Our study showed no significant difference between the pre- and post-treatment parameters for above-average lip thicknesses. The anteroposterior position change in the upper lip also proved to be significant compared to the E-line, which showed an increasing trend in the patients on average. This means that the upper lip was, on average, 1.93 mm ± 2.44 mm behind the E-line.

From the initial malocclusion point of view, a meta-analysis performed by Jason et al. concluded that in class II division 1 malocclusions, treated with premolar extractions, the NLA increased, and the lips were retracted [35]. The upper premolar extraction is mainly indicated in class II malocclusions and in upper arch crowding. In our study, all types of malocclusions were considered for treatment; therefore, further studies differentiating these aspects would be useful.

The limitations of the present study are mainly related to the number of subjects included in this study and to the specific treatment that was applied. Only upper premolars were extracted, so the influence of the lower premolar extraction was not considered. Another study limitation is that dental parameters like overjet and overbite were not measured.

Regarding the sample size calculation, only the main objective was considered. Further studies on the additional soft tissue features, such as lip competence and lip strain, which can influence the nasolabial parameters, would be helpful.

## 5. Conclusions

The upper and (Ls:SN) position changed significantly after the orthodontic treatment. In contrast, the position of the nasal bridge lip (Cm:SN) showed no significant changes. The factors measured during the cephalometric analysis, the soft tissue parameters, showed a significant change; thus, we can state that the orthodontic treatment influences the aesthetics of the facial profile. The repositioning of the upper lip is more acute when the thickness of the lip is decreased.

## Figures and Tables

**Table 1:** Landmarks and their definitions, as used in the cephalometric analysis.

Landmarks	Definitions
Sella—S	Center of the pituitary fossa of the sphenoid bone.
Nasion—N	Most anterior point on the frontonasal suture in the midsagittal plane.
Point A, subspinale—A	Deepest point of the curve of the anterior border of the maxilla.
Point B, submentale—B	Most posterior point in the concavity along the anterior border of the symphysis.
Subnasale—Sn	A sagittal point where the nasal septum and the upper lip meet in the midsagittal plane.
Columella nasi—Cm	The external end of the nasal septum.
Labral superior—Ls	The uppermost point of the upper lip.
Labral inferior—Ls_inf	The boundary of the lower lip and the skin intersected in the median sagittal plane.
Spina nasalis anterior—SNA	Most anterior midpoint of the anterior nasal spine of the maxilla.
Spina nasalis posterior—SNP	The sharp and well-defined posterior extremity of the nasal crest of the hard palate.
Incisor superior incisal—Is_i	The most anterior point on the labial surface of the most prominent maxillary central incisor.
Incisor superior apex—Is_a	The most apical point of the upper incisor root.
Pronasale—Mon	The most prominent point of the tip of the nose.
Soft Tissue Pogonion—Pog’	The most anterior point on the mandibular symphysis.
Gnathion—Gn	The most antero-inferior point on the mandibular symphysis.
Soft Tissue Gnathion—Gn’	
Gonion—Go	The most posterior and inferior point on the mandible corpus.

**Table 2:** Values of the nasolabial angle measured on the photographs (NLA_1) and radiographs (NLA_2).

	NLA_1 (° ± SD)	NLA_2 (° ± SD)
Before treatment	106.86° ± 12.88°	111.61° ± 11.97°
After treatment	107.97° ± 9.43°	113.42° ± 9.58°

**Table 3:** Skeletal, dental, and soft tissue parameters, before and after treatment.

Parameters	Before Treatment	After Treatment	*p* Value
SNA	80.75° ± 2.91°	79.98° ± 2.88°	0.06
SN-PP	8° ± 3.32°	8,15° ± 3.63°	0.22
ANS-Gn	115.59 mm ± 12.17 mm	116.86 mm ± 12.59 mm	0.08
GoGn-SN	148.67° ± 6.2°	149.61° ± 5.49°	0.62
Is-SN	103.75° ± 9.26°	102.18° ± 7.51°	0.41
Is-PP	111.74° ± 8.11°	110.33° ± 7.49°	0.44
Is-NA	23° ± 8.71°	22.2° ± 7.34°	0.99
Is-NA	7.84 mm ± 4.97 mm	6.43 mm ± 3.84 mm	0.18
ULT	21.4 mm ± 4.07 mm	22.9 mm ± 4.06 mm	0.03 *
UL-E	8.42 mm ± 4.84 mm	10.35 mm ± 423 mm	0.00 *

* Statistically significant.

## Data Availability

The data presented in this study are available on request from the corresponding author.
